# Using spike train distances to identify the most discriminative neuronal subpopulation

**DOI:** 10.1186/1471-2202-14-S1-P35

**Published:** 2013-07-08

**Authors:** Thomas Kreuz, Nebojsa Bozanic

**Affiliations:** 1 Institute for Complex Systems, CNR, Sesto Fiorentino, Italy

## 

During the last decade spike train distances [1-4] have become an essential means to characterize neural coding in a wide range of neurophysiological contexts. In a typical setup different stimuli are presented repeatedly and spike train distances are used to carry out a pairwise similarity analysis in order to evaluate whether responses to the same stimulus exhibit smaller distances than responses to different stimuli. With the increasing availability of multi-neuron recordings these kinds of analyses can now be performed not just for individual neurons but rather for simultaneously recorded neuronal populations.

Recently, the bivariate Victor-Purpura and the van Rossum distances [1,2] have been extended to quantify dissimilarities between multi-unit responses. These new population measures [5,6] have been designed such that they can estimate the discrimination performance of either the population as a whole ("summed population") or of individual neurons ("labelled line") or of interpolations between these two extremes. However, the question that these approaches fail to answer is the following: In cases the two extremes fail, which subpopulation within the larger population discriminates the presented stimuli best?

In this study we thus follow a complimentary approach and present an algorithm which addresses exactly this question. The brute-force approach of calculating the pairwise distance matrices and the stimulus discrimination performance for every possible neuronal subpopulation is not feasible even for moderate numbers of neurons. Instead, our algorithm relies on an iterative scheme which considerably restricts the number of subpopulations for which the pairwise distance matrices and the stimulus discrimination performance actually have to be calculated.

The spike train distance that we use to evaluate whether the responses elicited by different stimuli can be distinguished is the SPIKE-distance [4,7]. Whereas distances like the Victor-Purpura or the van Rossum spike train distance rely on a time-scale parameter, the SPIKE-distance is parameter-free and time-scale independent. This allows for easy comparability of results obtained for vastly different firing rates (which depend on the size of the pooled subpopulation). In contrast to the ISI-distance [3] it is sensitive to spike timing which is an important property that will be needed in some of the setups used.
